# TSG‐6 Is Weakly Chondroprotective in Murine OA but Does not Account for FGF2‐Mediated Joint Protection

**DOI:** 10.1002/acr2.11176

**Published:** 2020-10-07

**Authors:** Linyi Zhu, Shannah Donhou, Annika Burleigh, Jadwiga Miotla Zarebska, Marcia Curtinha, Ida Parisi, Sumayya Nafisa Khan, Francesco Dell’Accio, Anastasios Chanalaris, Tonia L. Vincent

**Affiliations:** ^1^ Kennedy Institute of Rheumatology Arthritis Research UK Centre for OA Pathogenesis University of Oxford UK; ^2^ William Harvey Research Institute Queen Mary University of London UK

## Abstract

**Objective:**

Tumor necrosis factor α–stimulated gene 6 (TSG‐6) is an anti‐inflammatory protein highly expressed in osteoarthritis (OA), but its influence on the course of OA is unknown.

**Methods:**

Cartilage injury was assessed by murine hip avulsion or by recutting rested explants. Forty‐two previously validated injury genes were quantified by real‐time polymerase chain reaction in whole joints following destabilization of the medial meniscus (DMM) (6 hours and 7 days). Joint pathology was assessed at 8 and 12 weeks following DMM in 10‐week‐old male and female fibroblast growth factor 2 (FGF2)^−/−^, TSG‐6^−/−^, TSG‐6^tg^ (overexpressing), FGF2^−/−^;TSG‐6^tg^ (8 weeks only) mice, as well as strain‐matched, wild‐type controls. In vivo cartilage repair was assessed 8 weeks following focal cartilage injury in TSG‐6^tg^ and control mice. FGF2 release following cartilage injury was measured by enzyme‐linked immunosorbent assay.

**Results:**

TSG‐6 messenger RNA upregulation was strongly FGF2‐dependent upon injury in vitro and in vivo. Fifteeen inflammatory genes were significantly increased in TSG‐6^−/−^ joints, including *IL1*α, *Ccl2*, and *Adamts5* compared with wild type. Six genes were significantly suppressed in TSG‐6^−/−^ joints including *Timp1, Inhibin* β*A*, and *podoplanin* (known FGF2 target genes). FGF2 release upon cartilage injury was not influenced by levels of TSG‐6. Cartilage degradation was significantly increased at 12 weeks post‐DMM in male TSG‐6^−/−^ mice, with a nonsignificant 30% reduction in disease seen in TSG‐6^tg^ mice. No differences were observed in cartilage repair between genotypes. TSG‐6 overexpression was unable to prevent accelerated OA in FGF2^−/−^ mice.

**Conclusion:**

TSG‐6 influences early gene regulation in the destabilized joint and exerts a modest late chondroprotective effect. Although strongly FGF2 dependent, TSG‐6 does not explain the strong chondroprotective effect of FGF2.

## INTRODUCTION

Tumor necrosis factor alpha (TNF‐α) stimulated gene 6 (TSG‐6) is a secreted product of TNF‐α–treated cells (1), which encodes for a 35 kDa multifunctional protein, consisting of Link and CUB (complement protein subcomponents C1r/C1s, urchin embryonic growth factor and bone morphogenetic protein 1) modules (2, 3, 4). TSG‐6 is expressed in response to a range of proinflammatory mediators (1, 2, 5) and is involved in a number of physiological processes including cervical ripening (6) and ovulation (7).

In murine models of inflammatory arthritis, TSG‐6 has been shown to protect the joint against damage. Delivery of recombinant protein led to a reduction in proteoglycan‐induced arthritis, whereas deletion of TSG‐6 in the same murine model led to increased severity of arthritis (8, 9). Chondroprotection was seen in mice overexpressing TSG‐6 in a collagen‐induced arthritis model (10) and in antigen‐induced arthritis wherein mice expressing a cartilage‐specific transgene of TSG‐6 had reduced aggrecan and cartilage degradation (11).

TSG‐6 has been detected at high levels in the synovial fluid of patients with rheumatoid arthritis and osteoarthritis (OA; 12, 13), and TSG‐6 levels are also highly elevated in human synovial fluid following joint injury (14). Moreover, TSG‐6 enzymatic activity, detected by the transfer of heavy chains from inter‐alpha‐inhibitor (IαI) to hyaluronan, has been identified as a biomarker for knee OA progression, such that increased TSG‐6 activity is associated with a higher risk of total knee replacement (15).

We have previously shown that TSG‐6 is strongly regulated in the mouse joint early following surgical destabilization of the medial meniscus (DMM), a model of OA. This regulation was deemed to be highly mechanosensitive as regulation was abrogated if the mice were prevented from mobilizing on their destabilized joint (16). We also showed that both in vivo and in vitro injury‐induced regulation of TSG‐6 was strongly dependent on fibroblast growth factor 2 (FGF2), a growth factor that is released from the cartilage matrix upon injury or loading (17, 18). Our previous work showed that mice deficient in FGF2 develop markedly accelerated OA upon surgical joint destabilization and upon ageing, indicating a chondroprotective role for FGF2 in vivo (19). FGF2 may be chondroprotective through its ability to suppress a disintegrin and metallopeptidase with thrombospondin type 1 motif 5 (ADAMTS5), one of the principal pathogenic aggrecan‐degrading enzymes in cartilage. Indeed, *Adamts5* was elevated in FGF2^−/−^ mice, and FGF2 was able to suppress interleukin (IL)‐1–induced aggrecanase activity in human articular cartilage explants in vitro (20). Whether this is a direct response or whether suppression of ADAMTS5 is through an intermediate protein, such as TSG‐6, is unknown.

In view of the strong anti‐inflammatory role of TSG‐6 in other arthritis models and its strong FGF2‐dependent gene regulation, in this paper we explore the hypothesis that the chondroprotective properties of FGF2 may in part be mediated through TSG‐6. We examine the course of disease after deletion or overexpression of TSG‐6 and ask whether TSG‐6 overexpression is able to prevent accelerated disease that is seen in FGF2^−/−^ mice.

## MATERIALS AND METHODS

#### Animals

**Figure 1:**
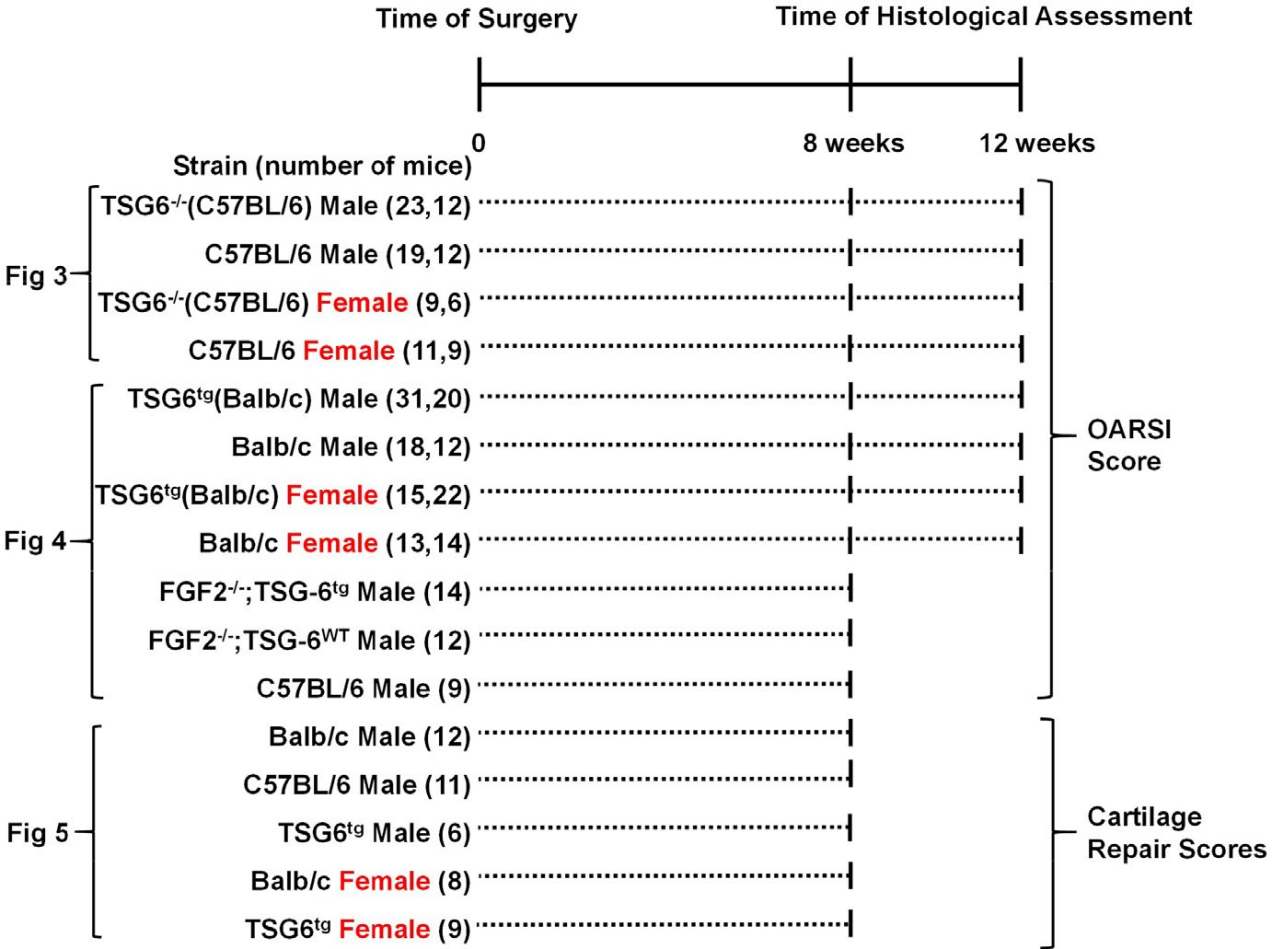
Summary of experiment design.

Animal experiments were carried out after gaining ethical approval in agreement with local policy. Four to six mice per cage were housed at 21°C in standard individually ventilated cages, maintained under a 12‐hour light/dark cycle. Mice were fed a certified mouse diet (RM3; Special Dietary Systems) and water ad libitum. TSG‐6 constitutive knockout (TSG‐6^−/−^) and cartilage‐specific (Col2‐driven, Balb/c background) constitutive overexpressing TSG‐6 transgenic mice (TSG‐6^tg^) (11) were obtained from Katalin Mikecz (Rush University Medical Center). TSG‐6^−/−^ animals were backcrossed onto a C57BL/6 background (for nine generations) and were bred as heterozygotes to generate wild‐type and knockout littermate controls. FGF2^−/−^ mice were originally purchased from Jackson Laboratory and were backcrossed onto a pure C57BL/6 background (nine generations). TSG‐6^tg^ (Balb/c) mice were crossed with FGF2^−/−^ (C57BL/6) to generate mixed litters of FGF2^−/−^;TSG‐6^tg^ and FGF2^−/−^;TSG‐6^WT^ (mixed background). Balb/c mice were obtained from Charles Rivers, United Kingdom laboratory. Figure 1 shows the schematic of the experiment design, which summarizes the in vivo experiments performed, including the total number of mice of each genotype in each experiment.

#### Surgical induction of OA

Ten‐week‐old mice were anesthetized by inhalation of isoflurane (3% induction and 1.5%‐2% maintenance) in 1.5 to 2 L/min oxygen. Following surgery, mice received a subcutaneous injection of Vetergesic (Alstoe Animal Health Limited). The mice were fully mobile 5 minutes after withdrawal of isoflurane. OA was induced by cutting the medial menisco‐tibial ligament as previously described (16). For sham surgeries, the joint was opened under anaesthesia but the menisco‐tibial ligament was left intact. Murine joints were harvested, and the skin and muscle bulk removed (16).

#### Histological assessment

Dissected knees were fixed in 10% formalin, decalcified in dilute formic acid, and embedded in paraffin. Coronal sections through the whole joint (80 µm apart) were cut and stained with Safranin O. Severity of cartilage destruction was assessed by a modified Osteoarthritis Research Society International OA grading system as previously described (19) by two blinded, independent scorers. At least eight sections were evaluated per joint. Results were expressed as the summed score, which was calculated by adding the three highest scores together from any given joint section (all four joint compartments). Osteophytes were scored by size (scores 0‐3) and maturity (scores 0‐3) (n = 5 per group) as previously described by Little et al (21).

#### Focal cartilage injury and histological assessment

Focal cartilage injury was carried out under a dissection microscope as previously described by Eltawil et al (22). Briefly, the joint was opened, the patella was dislocated, and a longitudinal full thickness injury was made in the patellar groove using the tip of a 25‐gauge needle. Patellar dislocation was reduced, and the joint capsule and skin were sutured closed. The contralateral knee was left as an unoperated control. Three transverse sections at 100‐µm intervals (22) were scored for cell morphology (scores 0‐4), matrix staining (scores 0‐4), filling of the defect (scores 0‐4), and osteochondral junction repair (scores 0‐2) according to the modified Pineda scale; a high score indicates better cartilage repair (scores were inverted in Eltawil et al (22)). Sums of these four categories were given for each section, and an average score was calculated (max score = 14).

#### Cartilage injury

Mice (5‐6 weeks old) were culled by carbon dioxide, and the acetabulofemoral (hip) joints were exposed by blunt dissection. The femoral cap (cartilage) was avulsed using forceps, as described previously (19). Murine hip cartilage was avulsed (avulsion injury) directly into serum‐free Dulbecco's Modified Eagle's Medium (DMEM) and incubated for 48 hours. Some explants were rested for 48 hours after avulsion and then either recut (cut with a scalpel into four pieces) or treated with FGF2 (250 ng/ml) in fresh DMEM and left for 4 hours prior to RNA extraction. For FGF2 release, cartilage explants (postavulsion) were immediately placed in 100‐µl serum‐free DMEM at 37°C for 30 minutes. The medium collected (injury‐conditioned medium) was stored at −80°C until FGF2 measurement.

#### FGF2 measurement in cartilage avulsion injury–conditioned medium

FGF2 levels in the injury‐conditioned medium were assayed in duplicate on single‐spot ultrasensitive V‐PLEX bFGF kit (catalog no. K151MDD) from Meso Scale Discovery (MSD). The assay was carried out according to the manufacturer’s instructions. Plates were read using MSD SPECTOR Imager 2400 measuring electrochemiluminescence. FGF2 concentrations were extrapolated from a standard curve calculated using a four‐parameter logistic fit using MSD Discovery Workbench software version 3.

#### RNA extraction and real‐time polymerase chain reaction

Four murine femoral heads were snap frozen and stored at −80°C. For whole joints, the joint was harvested at defined anatomical positions (patella insertion on tibia and quadriceps insertion on femur), and the skin and muscle removed as previously described (17). The femoral head cartilage or joints were pulverized using a PowerGen 125 Polytron instrument (Fisher Scientific), and RNA was extracted using Qiagen RNeasy Mini Kit according to the manufacturer’s instructions. RNA was reverse transcribed using a High‐Capacity cDNA kit (Applied Biosystems) and analyzed on 384‐well custom‐made TaqMan microfluidic cards.

#### Statistical analysis

Differences in gene expression levels between WT (C57BL/6) and TSG‐6^−/−^ joints were analyzed by unpaired multiple *t* tests and the *P* values corrected for multiplicity (*Q* values) using a family‐wise false discovery ratio of 5%. *Q* values are used in Table 1. Analysis of variance (ANOVA) with Sidak post hoc testing to adjust for multiplicity was used to compare TSG‐6 gene expression differences between WT and FGF2^−/−^ joints. Mann‐Whitney *U* test was used to analyze TSG‐6^−/−^ and TSG‐6^tg^ histological pathology scores. Where three genotypes were considered side by side (Figure 5), ANOVA was performed. Values of *P* < 0.05 were considered statistically significant. Spearman ρ correlation test was used to assess a relationship between fold gene expression and histological score. Where data were not normally distributed (tested using the D’Agostino normality test), and in the case of nonparametric data like osteophyte and focal cartilage data, Mann‐Whitney tests were used. Statistical testing was performed using GraphPad Prism 7 and SPSS 26.0 (IBM) software.

**Table 1:** Gene expression profiles of whole joint in TSG‐6^−/−^ and WT following DMM.

	0h (Mean Fold of Change ± SEM)			6h/0h (Mean Fold of Change ± SEM)			7d/0h (Mean Fold of Change ± SEM)		
Gene ID	WT	TSG‐6^−/−^	*Q* value	WT	TSG‐6^−/−^	*Q* value	WT	TSG‐6^−/−^	*Q* value
***Aggrecan***	1.00 ± 0.06	1.03 ± 0.03	0.6424	0.89 ± 0.08	1.02 ± 0.12	0.4529	0.71 ± 0.02	0.81 ± 0.11	0.3972
***Adam8***	1.07 ± 0.17	1.11 ± 0.09	0.8616	2.41 ± 0.06	3.70 ± 0.33	0.018	1.31 ± 0.02	3.01 ± 0.24	0.0022
***Adam9***	1.12 ± 0.08	1.10 ± 0.09	0.8712	2.22 ± 0.07	2.84 ± 0.24	0.065	2.20 ± 0.11	1.47 ± 0.17	0.0232
***Adamts1***	0.98 ± 0.12	1.04 ± 0.05	0.6512	7.25 ± 1.54	2.40 ± 0.20	0.0351	3.10 ± 0.58	0.27 ± 0.04	0.008
***Adamts15***	0.98 ± 0.02	1.17 ± 0.09	0.1026	12.70 ± 0.65	13.54 ± 0.94	0.5087	7.34 ± 0.67	13.66 ± 0.71	0.0029
***Adamts4***	1.21 ± 0.12	1.14 ± 0.07	0.6099	3.90 ± 0.26	4.42 ± 0.34	0.2941	1.87 ± 0.22	2.19 ± 0.09	0.2514
***Adamts5***	1.17 ± 0.09	1.09 ± 0.11	0.6417	1.50 ± 0.13	3.10 ± 0.10	0.0006	1.57 ± 0.21	2.35 ± 0.15	0.042
***Arginase 1***	0.96 ± 0.10	0.82 ± 0.11	0.3997	84.23 ± 4.06	309.06 ± 32.50	0.0024	3.14 ± 1.05	317.81 ± 13.19	<0.0001
***Arginase 2***	1.15 ± 0.12	1.20 ± 0.08	0.7661	1.05 ± 0.01	13.72 ± 0.89	0.0001	1.15 ± 0.09	13.29 ± 0.71	<0.0001
***Ccl2***	1.09 ± 0.08	1.03 ± 0.02	0.494	131.41 ± 10.93	239.79 ± 8.64	0.0015	20.90 ± 0.64	29.31 ± 2.56	0.0332
***Ccl5***	1.03 ± 0.02	1.19 ± 0.12	0.2392	1.52 ± 0.07	1.97 ± 0.06	0.0081	0.73 ± 0.30	2.21 ± 0.81	0.163
***Ccl7***	0.96 ± 0.03	1.04 ± 0.03	0.1759	5.57 ± 1.93	10.04 ± 2.48	0.228	2.33 ± 0.29	18.31 ± 2.68	0.0041
***Ccr2***	0.75 ± 0.14	0.98 ± 0.46	0.654	1.11 ± 0.04	1.21 ± 0.10	0.4285	1.41 ± 0.26	2.38 ± 0.64	0.2298
***Ccr5***	1.21 ± 0.11	1.04 ± 0.16	0.428	3.50 ± 0.15	11.44 ± 0.79	0.0006	1.32 ± 0.15	19.71 ± 3.24	0.0048
***Cd14***	0.94 ± 0.16	1.09 ± 0.19	0.5612	5.16 ± 0.72	8.34 ± 0.51	0.0228	1.89 ± 0.51	2.90 ± 0.12	0.1236
***Cd68***	1.12 ± 0.34	0.65 ± 0.10	0.252	2.14 ± 0.05	4.98 ± 0.90	0.0345	1.35 ± 0.17	3.18 ± 0.33	0.0083
***Col2a1***	1.11 ± 0.09	1.10 ± 0.03	0.9419	0.31 ± 0.04	0.43 ± 0.17	0.5567	0.19 ± 0.04	0.34 ± 0.12	0.3177
***Ctgf***	0.74 ± 0.21	0.27 ± 0.13	0.1327	0.20 ± 0.05	0.35 ± 0.12	0.3289	0.17 ± 0.04	0.44 ± 0.18	0.2205
***F3***	1.10 ± 0.09	1.00 ± 0.14	0.6016	1.13 ± 0.09	12.94 ± 0.25	<0.0001	2.14 ± 0.14	2.82 ± 0.13	0.0224
***Has1***	1.21 ± 0.11	1.15 ± 0.18	0.7819	3.73 ± 0.26	8.70 ± 0.55	0.0013	0.57 ± 0.29	3.07 ± 0.87	0.052
***Has2***	1.08 ± 0.08	1.05 ± 0.04	0.7588	2.04 ± 0.04	3.24 ± 0.69	0.1542	2.35 ± 0.34	2.64 ± 0.10	0.4547
***Il1a***	1.07 ± 0.04	1.04 ± 0.03	0.5471	1.21 ± 0.06	2.71 ± 0.09	0.0002	1.02 ± 0.01	2.51 ± 0.22	0.0026
***Il1b***	1.00 ± 0.00	1.16 ± 0.08	0.1215	5.17 ± 0.16	5.31 ± 0.65	0.8441	1.92 ± 0.21	2.77 ± 0.56	0.2268
***Il1r1***	1.18 ± 0.12	0.86 ± 0.19	0.2308	3.65 ± 0.39	7.07 ± 0.42	0.0039	1.31 ± 0.04	2.24 ± 0.35	0.0559
***Il1rl1***	1.03 ± 0.02	1.21 ± 0.10	0.1517	2.02 ± 0.16	2.11 ± 0.15	0.6776	1.47 ± 0.22	1.45 ± 0.18	0.9486
***Il33***	1.09 ± 0.05	1.30 ± 0.01	0.0127	3.20 ± 0.26	5.48 ± 0.32	0.0052	1.70 ± 0.35	4.53 ± 0.09	0.0014
***Il6***	1.25 ± 0.13	1.16 ± 0.25	0.7802	16.84 ± 1.67	50.02 ± 11.37	0.0447	0.99 ± 0.33	123.72 ± 14.94	0.0012
***Inhba***	1.06 ± 0.17	0.82 ± 0.09	0.2862	2.73 ± 0.05	0.93 ± 0.27	0.0027	0.70 ± 0.26	0.82 ± 0.09	0.6733
***Mmp13***	0.96 ± 0.02	1.50 ± 0.33	0.1815	0.42 ± 0.04	4.48 ± 1.67	0.0721	0.61 ± 0.24	6.92 ± 2.29	0.0519
***Mmp19***	1.17 ± 0.11	0.93 ± 0.23	0.3946	3.36 ± 0.25	3.56 ± 0.11	0.5015	1.82 ± 0.20	5.86 ± 1.68	0.0756
***Mmp3***	1.36 ± 0.18	1.26 ± 0.26	0.7757	6.01 ± 0.00	3.21 ± 0.30	0.0008	2.01 ± 0.00	9.84 ± 0.34	<0.0001
***Mmp8***	1.19 ± 0.05	1.03 ± 0.01	0.0286	2.04 ± 0.02	3.69 ± 0.82	0.1137	1.01 ± 0.00	3.43 ± 1.25	0.125
***Nos2***	1.00 ± 0.00	1.16 ± 0.14	0.3365	14.73 ± 1.86	25.34 ± 3.53	0.0566	8.18 ± 0.74	21.75 ± 1.83	0.0023
***Pdpn***	0.97 ± 0.15	0.98 ± 0.19	0.9671	6.87 ± 0.22	2.09 ± 0.82	0.0048	2.30 ± 0.21	1.38 ± 0.16	0.0256
***Ptges***	0.85 ± 0.15	1.20 ± 0.08	0.1083	1.37 ± 0.09	1.99 ± 0.01	0.0021	1.25 ± 0.25	2.58 ± 0.40	0.0495
***Ptgs2***	1.09 ± 0.06	1.05 ± 0.01	0.5115	14.40 ± 1.41	21.76 ± 2.12	0.0443	9.04 ± 1.05	23.83 ± 2.28	0.0042
***Sfrp2***	0.92 ± 0.19	1.05 ± 0.02	0.5473	0.58 ± 0.22	2.93 ± 0.56	0.0171	3.11 ± 0.45	3.44 ± 0.37	0.5949
***Timp1***	0.93 ± 0.09	0.44 ± 0.01	0.0068	4.90 ± 0.26	1.57 ± 0.04	0.0002	1.24 ± 0.16	1.78 ± 0.28	0.1737
***Tnfrsf12a***	1.05 ± 0.07	0.83 ± 0.14	0.243	5.83 ± 0.30	1.08 ± 0.15	0.0001	2.58 ± 0.23	1.12 ± 0.12	0.0047
***TSG‐6***	1.14 ± 0.14			31.19 ± 0.13			45.27 ± 1.17		
***Wisp2***	0.96 ± 0.05	1.09 ± 0.07	0.1775	7.03 ± 0.24	6.66 ± 0.47	0.5142	4.24 ± 0.23	3.08 ± 0.29	0.034
***Wnt16***	0.92 ± 0.11	0.88 ± 0.10	0.7704	0.95 ± 0.04	0.44 ± 0.03	0.0007	0.79 ± 0.15	0.73 ± 0.17	0.8061

Note

The effect of TSG‐6 deletion on relative mRNA levels for injury response genes in whole murine joints of WT (C57BL/6) and TSG‐6^−/−^ mice (n = 3 mice per group) 0 hours, 6 hours, or 7 days post‐DMM. Values are the mean fold change ± SEM. Results are expressed relative to *18s*. Statistical significance was determined using unpaired multiple *t* tests and the *P* values corrected for multiplicity (*Q* values) using a discovery ratio of 5%. Genes highlighted were shown to be significant (light grey = increase, dark grey = decrease).

Abbreviations: *Adam*, a disintegrin and metalloproteinase; *Adamts*, a disintegrin and metalloproteinase with thrombospondin motifs; *Ctgf*, connective tissue growth factor; DMM, destabilization of the medial meniscus; *F3*, coagulation factor III; *Has1*, hyaluronan synthase 1; Inhba, inhibin beta A; *Nos*, nitric oxide synthase; *Pdpn*, podoplanin; *Ptges*, prostaglandin E synthase; *Ptgs2*, prostaglandin‐endoperoxide synthase 2; *Sfrp2*, secreted frizzled‐related protein 2; *Timp1*, tissue inhibitor of metalloproteinase 1; *Tnfrsf12a*, tumor necrosis factor receptor superfamily member 12A (TWEAK receptor); TSG‐6, tumor necrosis factor α–stimulated gene 6; *Wisp2*, WNT1‐inducible‐signaling pathway protein 2; WT, wild type.

## RESULTS

#### TSG‐6 is induced upon injury in an FGF2‐dependent manner

**Figure 2:**
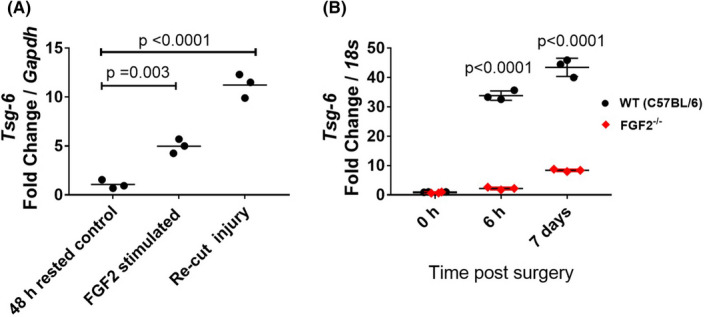
Tumor necrosis factor α–stimulated gene 6 (TSG‐6) is regulated in an fibroblast growth factor 2 (FGF2)‐dependent manner. **A**, Regulation of TSG‐6 gene expression by recutting injury or FGF2 stimulation of murine cartilage explants. Murine hip cartilage (from male animals) was avulsed (avulsion injury) directly into serum‐free Dulbecco's Modified Eagle's Medium (DMEM) and incubated for 48 hours (48 h). Rested cartilage was either recut (recut injury) or treated with FGF2 (250 ng/ml) in fresh DMEM and left for 4 hours prior to RNA extraction. Each point represents the messenger RNA (mRNA) from four hips pooled. *Tsg6* levels were expressed relative to *Gapdh* and normalized to the control. **B**, RNA was extracted from male wild‐type (WT) or FGF2^−/−^ mouse joints (n = 3) at specified times after destabilization of the medial meniscus. *Tsg6* mRNA levels were expressed relative to *18s* and normalized to the WT 0 h control. Statistical significance was determined by analysis of variance with Sidak post hoc testing, comparing treated compared with rested controls (**A**) and WT with FGF2^−/−^ (**B**) for each time point.

We first confirmed TSG‐6 regulation following cartilage injury in vivo and in vitro as previously shown by our group (). TSG‐6 was strongly induced upon recutting injury or FGF2 stimulation (Figure 2A). It was also strongly upregulated in whole joints after DMM in an FGF2‐dependent manner (Figure 2B); upregulation of TSG‐6 was suppressed in FGF2^−/−^ mice.

#### Deletion of TSG‐6 leads to increased inflammatory genes and decreased FGF2‐dependent gene regulation following DMM

We next wanted to see how TSG‐6 influenced the regulation of inflammatory response genes in the whole joint following DMM. TaqMan microfluidic cards were prepared for a number of known inflammatory and FGF2‐dependent genes previously found to be upregulated in whole joints following DMM or after in vitro cartilage injury (16, 17). Twenty‐eight genes were significantly regulated at any point postsurgery compared with the 0‐hour unoperated controls. Fifteen genes (Table 1) were significantly upregulated in TSG‐6**^−/−^** joints compared with WT (C57BL/6) joints 6 hours after DMM, including inflammatory response genes such as *Adamts5*, *Ccl2*, and *IL‐1α*. These findings appear consistent with published studies showing an anti‐inflammatory effect of TSG‐6. Six genes were significantly suppressed in TSG‐6^−/−^ joints compared with WT (C57BL/6) joints 6 hours after DMM, including *Inhibin βA*, *Tnfrsf12a*, *Podoplanin*, and *Timp1*. Interestingly, five out of the six genes that were strongly suppressed in TSG‐6^−/−^ joints had previously been shown to be highly FGF2 dependent in vivo and in vitro (17).

#### Male TSG‐6^−/−^ mice show a modest late increase in disease following DMM

**Figure 3:**
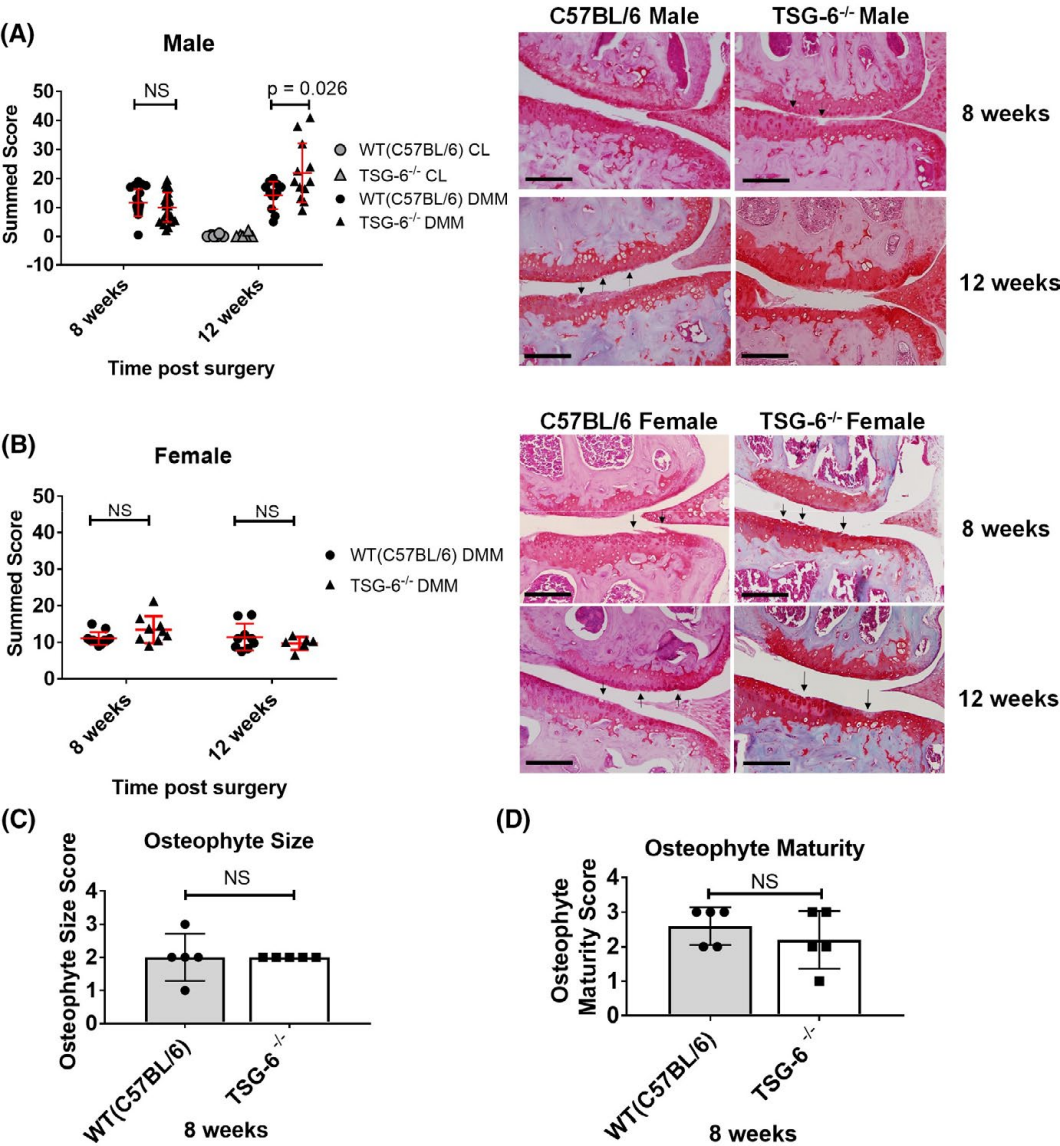
Increased cartilage degradation in male tumor necrosis factor α–stimulated gene 6 (TSG‐6^−/−^) mice 12 weeks after destabilization of the medial meniscus (DMM). Histological chondropathy scores and representative histologic sections of knee cartilage 8 or 12 weeks after DMM in male (**A**) and female (**B**) wild‐type (WT; C57BL/6) and TSG‐6^−/−^ mice. Black arrows in the images indicate cartilage defects. Scale bar = 200 µm. Statistical significance was determined by Mann‐Whitney *U* tests. Mean osteophyte scores: size (**C**) and maturity (**D**) in WT (C57BL/6) and TSG‐6^−/−^ male mice 8 weeks post‐DMM. Also shown are contralateral (CL) joint controls. NS, not significant.

Gene expression profiles suggested an increase in inflammatory mediators in the joint associated with a reduction of FGF2‐dependent genes. As FGF2 has been shown to be chondroprotective (19), we hypothesized that TSG‐6^−/−^ mice might develop accelerated OA following DMM. We examined the susceptibility of 10‐week‐old male and female WT (C57BL/6) and TSG‐6^−/−^ mice to DMM‐induced OA and compared the summed scores 8 and 12 weeks after surgery. No differences were seen between TSG‐6^−/−^ and WT (C57BL/6) mice in either males or females at 8 weeks post‐DMM (Figure 3A and B). At 12 weeks post‐DMM, however, TSG‐6^−/−^ male mice, but not female mice, had a statistically significant 50% increase in mean disease score (21.9 ± 10.1) compared with WT (C57BL/6) DMM controls (14.2 ± 4.7) (Figure 3A). No disease was seen in the contralateral joints (of DMM operated mice) of either genotype at either time point. Osteophytes are established early (from 1 week) post‐DMM (23). At 8 weeks post‐DMM, there were no significant changes in osteophyte size or maturity in male mice (Figure 3C and D). This was also the case at 12 weeks (data not shown).

#### Male transgenic mice show a nonsignificant 30% reduction in disease

**Figure 4:**
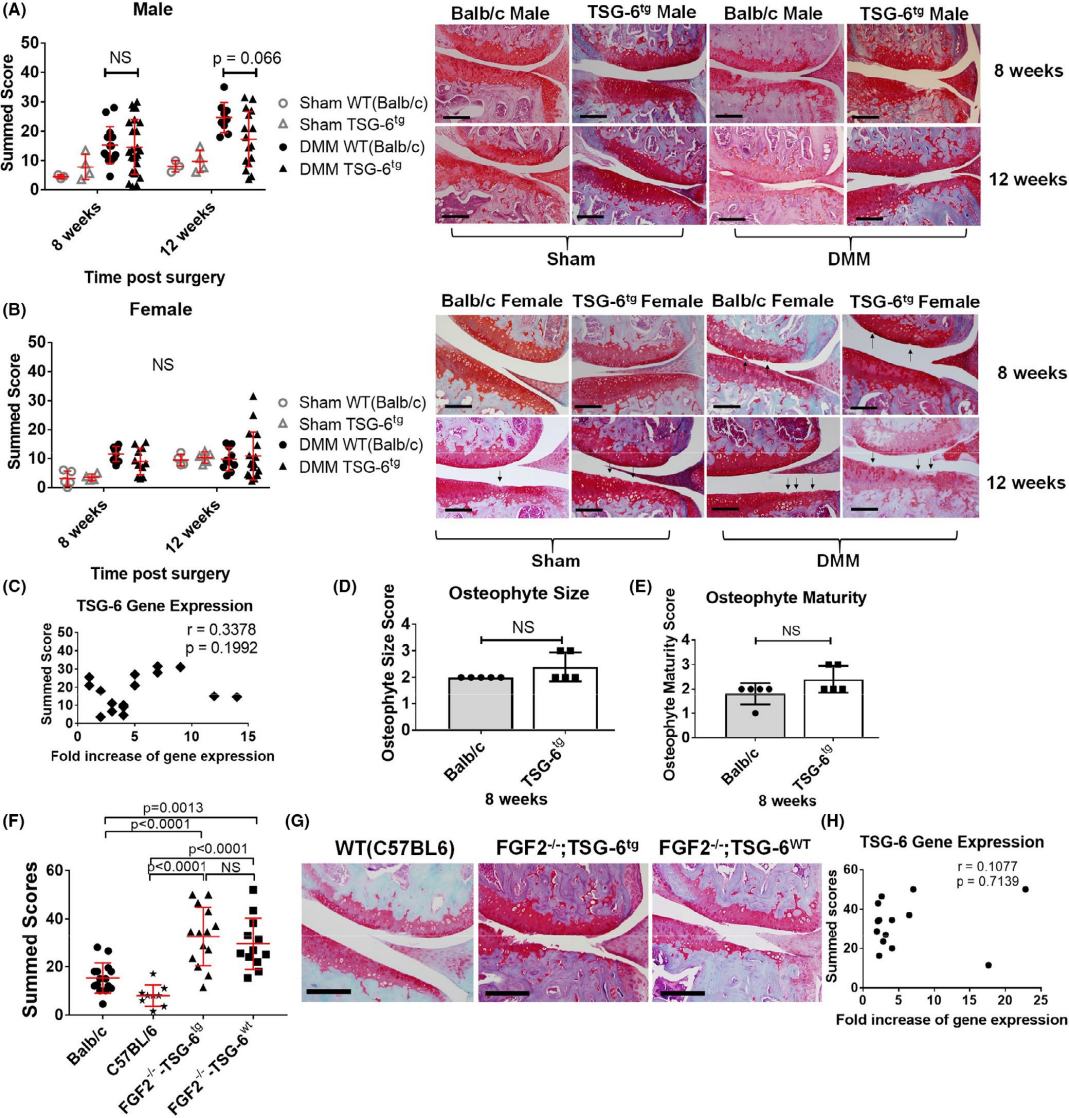
Joint pathology in tumor necrosis factor α–stimulated gene 6 (TSG)^tg^ mice at 8 and 12 weeks after destabilization of the medial meniscus (DMM). Histological chondropathy scores (left) and representative histologic sections (right) of knee cartilage 8 and 12 weeks after sham or DMM in male (**A**) and female (**B**) TSG‐6^tg^ and wild‐type (WT; Balb/c) mice. Black arrows in the images indicate cartilage defects. Scale bar = 200 µm. Statistical significance was determined by Mann‐Whitney *U* tests. There were no significant differences seen between any of the female experimental groups by two‐way analysis of variance (ANOVA). **C**, Fold increase of *Tsg6* over WT transgene (by quantitative reverse transcription polymerase chain reaction) was plotted against summed cartilage score for male mice 12 weeks post‐DMM. *R* ^2^ correlation statistical analysis was performed. Mean osteophyte scores, size (**D**) and maturity (**E**), were carried out in WT (Balb/c) and TSG‐6^tg^ male mice 8 weeks after DMM. Histological scores (left) (**F**) and representative histologic sections (right) (**G**) of knee cartilage 8 weeks after DMM in male WT(C57BL6), fibroblast growth facror 2 (FGF2)^−/−^;TSG‐6^tg^, and FGF2^−/−^;TSG‐6^WT^ mice. Balb/c histology scores are derived from Figure 4A. Statistical significance was determined by one‐way ANOVA with Sidak post hoc testing. **H**, Level of *Tsg6* expression was plotted against summed cartilage score for male FGF2^−/−^;TSG‐6^tg^ mice 8 weeks post‐DMM. R^2^ correlation statistical analysis was performed.

We next examined the susceptibility of male and female TSG‐6^tg^ mice following DMM. Cartilage degradation was assessed by histology 8 and 12 weeks after DMM. Male TSG‐6^tg^ mice showed a 30% reduction in mean disease score at 12 weeks post‐DMM that did not reach statistical significance after correcting for multiple testing (*P* = 0.066, Mann‐Whitney *U* test) (Figure 4A). No differences were observed in sham‐operated joints between genotypes at either 8 or 12 weeks postsurgery. There were no significant differences between any of the female experimental groups (Figure 4B). As male mice showed a nonsignificant reduction in disease mean score at 12 weeks post‐DMM, we looked at the amount of transgene expressed and whether this correlated with the cartilage degradation score. There was no correlation between the amount of transgene expressed (in the contralateral joint) and the severity of cartilage damage 12 weeks post‐DMM (Figure 4C), nor was there a difference in osteophyte size or maturity in male mice 8 weeks post‐DMM (Figure 4D and E).

In order to see whether TSG‐6 might be mediating the protection afforded by FGF2, we tested whether overexpression of TSG‐6 would compensate for loss of FGF2. TSG‐6^tg^ mice were crossed with FGF2^−/−^ mice to generate mixed litters of FGF2^−/−^;TSG‐6^tg^ and FGF2^−/−^;TSG‐6^WT^ mice (on a mixed background). Deletion of FGF2 (FGF2^−/−^;TSG‐6^WT^) led to severe disease compared with WT C57BL/6 or Balb/c mice consistent with our previous publication (19). Overexpression of TSG‐6 (FGF2^−/−^;TSG‐6^tg^) was unable to compensate for loss of FGF2 (Figure 4F and G), and disease scores did not correlate with transgene level (Figure 4H).

#### Overexpression of TSG‐6 does not affect healing of focal cartilage defects

**Figure 5:**
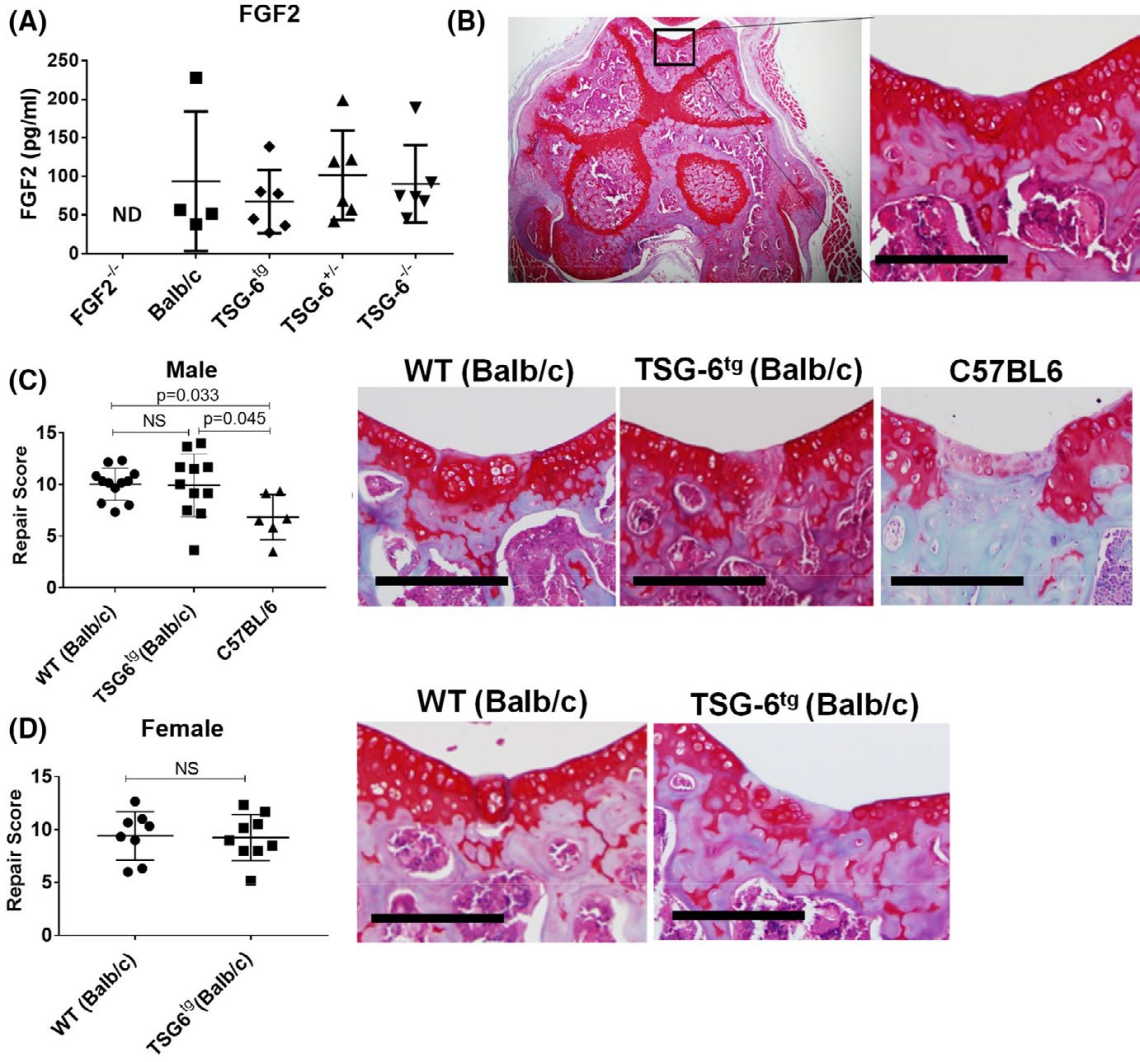
Tumor necrosis factor α–stimulated gene 6 (TSG‐6)^tg^ mice do not have enhanced cartilage repair capacity. **A**, fibroblast growth factor 2 (FGF2) levels were measured by enzyme‐linked immunosorbent assay (MSD PLEX bFGF assay) in conditioned medium collected from injured mouse hips (a mixture of male and female) from wild‐type (WT; Balb/c), TSG‐6^tg^, TSG‐6^+/‐^ (heterozygotes), and TSG‐6^−/−^ (knockout, homozygotes) mice. **B**, Transverse section of the joint showing the position of patella groove. **C**, Cartilage repair scores (left) and representative histological images of Safranin O–stained sections (right) 8 weeks after focal cartilage injury in male WT (Balb/c mice), TSG‐6^tg^, and C57BL/6 mice, n = 6 to 12 mice per group. **D**, Cartilage repair scores after focal cartilage injury in female WT (Balb/c) and TSG‐6^tg^ mice. Scale bar = 200 µm. Mann‐Whitney *U* tests were used to determine statistical significance.

Chondroprotection may be mediated by enhanced cartilage repair within the joint. We considered whether TSG‐6 affected the bioavailability of FGF2, a repair cytokine, after articular cartilage injury, and whether overexpression of TSG‐6 would influence the healing of cartilage in vivo. To address the former, conditioned medium from injured mouse hips from FGF2^−/−^, WT (Balb/c), TSG‐6^+/‐^ (heterozygotes), TSG‐6^−/−^ (homozygotes), and TSG‐6^tg^ mice were assayed for FGF2 (Figure 5A). Levels of FGF2 were equivalent between groups (apart from that generated by FGF2^−/−^ cartilage). These results suggested that the bioavailability of FGF2 after injury was not determined by TSG‐6 levels. Next, we generated full thickness defects in the patellar groove of TSG‐6^tg^, WT (Balb/c), and C57BL/6 mice using a model of cartilage regeneration that has been shown to be strain and age dependent (22). Ten‐week‐old male and female Balb/c mice produced superior repair compared with C57BL/6 controls 8 weeks after surgery (Figure 5C and D). No difference in repair was seen between TSG‐6^tg^ and WT (Balb/c) in either male or female mice.

## DISCUSSION

TSG‐6 messenger RNA (mRNA) is strongly upregulated post‐DMM and has a major influence on suppressing inflammatory genes as well as influencing FGF2‐dependent genes early following induction of OA. In vivo data demonstrated increased cartilage degradation 12 weeks post‐DMM in male TSG‐6^−/−^ mice and a nonsignificant 30% reduction of mean disease score in TSG‐6^tg^ mice at the same time point (12 weeks). These data collectively suggest that TSG‐6 has a real, albeit modest chondroprotective role in the joint. Despite its strong FGF2 dependence, TSG6 overexpression appeared to be unable to reverse accelerated disease seen in FGF2^−/−^ mice indicating that it is not the principal driver of FGF2‐dependent chondroprotection. It was possible that the increase in mRNA did not translate to an increase in protein, but we were unable to validate this by immunohistochemistry (data not shown). When we considered gender, female mice developed very modest disease post‐DMM, as previously shown (24). Little disease progression was seen over time in these mice and there was no detectable effect of genotype.

The function of TSG‐6 remains poorly understood, and it remains unclear whether modest joint protection is afforded by its anti‐inflammatory or other actions. The anti‐inflammatory actions of TSG‐6 may be related to the ability of the Link module of TSG‐6 to bind to chemokines from the CXC and CC families (25), inhibiting neutrophil migration (26, 27, 28) or interfering with their matrix‐binding partners heparin and heparan sulfate (HS) (29, 30). Murine OA induced by DMM is characterized by transient synovitis, which is apparent immediately postsurgery, but little overt neutrophilic infiltration is seen beyond 2 weeks (T.L. Vincent: unpublished data). We did not attempt to measure levels of inflammatory cells in the OA joints, although there were increases in leukocyte markers such as CD14 and CD68 in whole‐joint extracts of TSG‐6^−/−^ compared with WT (C57BL/6) animals early post‐DMM. We also did not specifically assess synovitis by histological scoring as this is difficult to do using coronal joint sections. Other inflammatory genes that were upregulated in the TSG‐6^−/−^ joints included cytokines such as *Il1α*, *Ccl2*, and *Il6*. Although these molecules have proposed procatabolic actions in the joint, they are probably being made by nonleukocytic cells, eg, chondrocytes, and published, as well as unpublished data from our group, do not support a role for these molecules in driving disease (31, 32). If TSG‐6 is not acting by inhibiting leukocyte migration to suppress OA, it may be controlling cartilage loss by downregulating the protease network (33, 34). This is partly mediated by the formation of a complex between the Link module of TSG‐6 with IαI (35), a serine protease inhibitor. The inhibitory effect of this complex is specific for plasmin, a key activator of several MMPs, that is induced in murine OA by direct mechanical injury (16).

Our data show that TSG‐6 promotes several FGF2‐dependent genes with putative anti‐inflammatory/repair functions (eg, the tissue inhibitor of metalloproteinase, *Timp1* and *inhibin βA*, the dimer of which forms *activin A* [a TGFβ family member]). These genes are strongly induced in vivo and in vitro upon cartilage injury (17) and have purported chondroprotective actions by anticatabolic and prorepair roles. The fact that they are also TSG‐6 dependent suggests either that TSG‐6 can influence these genes directly (by an unknown mechanism) or that TSG‐6 affects the regulation or bioavailability of FGF2. We speculated that TSG‐6 could be influencing the binding of FGF2 in the pericellular matrix of cartilage, where it resides and is released upon tissue injury (36, 37). However, the latter did not appear to be the case as the release of FGF2 upon cartilage injury was not influenced by the level of TSG‐6 expression. Other complex actions of TSG‐6 have been described, such as heavy chain transfer–mediated stabilization of the extracellular matrix (38, 39) and interference of tissue‐derived morphogenetic proteins such as BMP2 (40), which could possibly account for the influence that we are describing.

In the past decade, interest has turned to the role of TSG‐6 in mesenchymal stem cells (MSCs); secreted TSG‐6 is thought to mediate their immunomodulatory and tissue‐protective properties (41, 42). TSG‐6, as well as FGF2, regulate morphology and crucial cellular processes for the maintenance of stemness and biological properties of MSCs (43, 44). However, if the principal role of TSG‐6 is to act on MSCs to enhance their repair capacity, then we should have expected to see a change in repair score after focal cartilage injury. Our results show overexpression of TSG‐6 has no influence on this repair. The focal cartilage injury model has not previously been explored in Balb/c mice and shows that this strain repairs well, in a similar fashion to DBA/1 mice (22). To fully exclude a prorepair action of TSG‐6, it would be necessary to perform the focal cartilage defect in transgenic mice backcrossed onto a nonrepairing strain, such as C57BL/6. This is beyond the scope of the current project.

TSG‐6 activity, measured by heavy chain transfer, has been described as a biomarker for disease progression and is associated with increases in other inflammatory mediators, including TIMP1, MMP3, and IL‐6 (15, 45). Our data do not support a pro‐disease role for TSG‐6 in OA, which suggests that correlation with disease progression may be epiphenomenal rather than causal. This is probably also the case following an acute knee injury, where synovial fluid TSG‐6 levels follow a similar pattern to several other inflammatory response proteins (14), which in part reflects the severity of the injury. Despite considerable efforts by a number of groups, the precise mechanism of action of TSG‐6 remains elusive and its therapeutic potential in OA, speculative.

This study did have limitations. We did not perform in‐depth analysis of the bone in TSG‐6^−/−^ mice. Deletion of TSG‐6 has been shown to influence bone microarchitecture by modulating both osteoblast and osteoclast function, which could potentially affect the biomechanical response in the joint following DMM (40, 46). This is unlikely to have influenced the TSG‐6 overexpressing mice as overexpression was driven by Type II collagen in these mice, and the effects should be more restricted to the articular cartilage. Neither did we perform synovitis scoring nor pain assessments on these mice because of limitations imposed by coronal sectioning of the joints, which we routinely perform for OA cartilage scoring. Because of poor breeding of Tsg6^+/‐^ (heterozygotes), we were limited by the number of animals available and did not perform sham operations in this strain or examine the effect of genotype with age, which in our experience requires much larger numbers. We recognize, as discussed above, the limitation of examining focal cartilage repair in Balb/c mice when they already appear to have moderately good intrinsic repair capability (not known at the start of our experiment). We also recognize the difficulties of trying to make conclusions from data that either just succeed or just fail to reach statistical significance after stringent correction for multiple testing. We powered this study to detect a 40% change in disease score between genotypes at any time point. A retrospective power calculation indicates that we needed four additional wild‐type mice (n = 16 WT, n = 20 TSG‐6^tg^, at 12 weeks post‐DMM) for the 30% reduction in disease to reach statistical significance.

## AUTHOR CONTRIBUTIONS

All authors were involved in drafting the article or revising it critically for important intellectual content, and all authors approved the final version to be published. Prof. Vincent has full access to all of the data in the pooled study and takes responsibility for the integrity of the data and the accuracy of the data analysis.

### Study conception and design

Zhu, Donhou, Burleigh, Zarebska, Curtinha, Parisi, Khan, Dell’Accio, Chanalaris, Vincent.

### Acquisition of data

Zhu, Donhou, Burleigh, Zarebska, Curtinha, Parisi, Khan, Chanalaris.

### Analysis and interpretation of data

Zhu, Donhou, Burleigh, Zarebska, Curtinha, Parisi, Khan, Dell’Accio, Chanalaris, Vincent.
